# ELISA reagent coverage evaluation by affinity purification tandem mass spectrometry

**DOI:** 10.1080/19420862.2017.1349586

**Published:** 2017-07-14

**Authors:** Scott M. Henry, Elissa Sutlief, Oscar Salas-Solano, John Valliere-Douglass

**Affiliations:** Seattle Genetics, Inc., Bothell, WA, USA

**Keywords:** Affinity purification, ELISA coverage, HCP, immunoprecipitation, mass spectrometry, MS

## Abstract

Host cell proteins (HCPs) must be adequately removed from recombinant therapeutics by downstream processing to ensure patient safety, product quality, and regulatory compliance. HCP process clearance is typically monitored by enzyme-linked immunosorbent assay (ELISA) using a polyclonal reagent. Recently, mass spectrometry (MS) has been used to identify specific HCP process impurities and monitor their clearance. Despite this capability, ELISA remains the preferred analytical approach due to its simplicity and throughput. There are, however, inherent difficulties reconciling the protein-centric results of MS characterization with ELISA, or providing assurance that ELISA has acceptable coverage against all process-specific HCP impurities that could pose safety or efficacy risks. Here, we describe efficient determination of ELISA reagent coverage by proteomic analysis following affinity purification with a polyclonal anti-HCP reagent (AP-MS). The resulting HCP identifications can be compared with the actual downstream process impurities for a given process to enable a highly focused assessment of ELISA reagent suitability. We illustrate the utility of this approach by performing coverage evaluation of an anti-HCP polyclonal against both an HCP immunogen and the downstream HCP impurities identified in a therapeutic monoclonal antibody after Protein A purification. The overall goal is to strategically implement affinity-based mass spectrometry as part of a holistic framework for evaluating HCP process clearance, ELISA reagent coverage, and process clearance risks. We envision coverage analysis by AP-MS will further enable a framework for HCP impurity analysis driven by characterization of actual product-specific process impurities, complimenting analytical methods centered on consideration of the total host cell proteome.

## Introduction

Host cell proteins (HCPs) must be adequately cleared from recombinant proteins by downstream processing to ensure patient safety, product quality, and regulatory compliance. Typically, HCP clearance is monitored by enzyme-linked immunosorbent assay (ELISA) since these assays provide a semi-quantitative measure of total HCP levels, high throughput, and are amenable to implementation in a regulated testing environment.^1-3^ A critical component of an HCP ELISA is a polyclonal anti-HCP reagent obtained from an immunization campaign. This reagent can be commercially sourced, process-specific, or product-specific, depending on the source of the antigen used in the immunization campaign and the stage of clinical development. In all cases, it is critical that the reagent has suitably comprehensive immunoreactivity (coverage) against the HCP impurities to enable effective monitoring of process clearance. HCP immunoreactivity is assessed by techniques such as 2 dimensional (2D)-Western blot or 2D-difference in gel electrophoresis (2D-DIGE) as a means to compare the total HCP population found in production harvest fluids with the subset recognized by the ELISA reagent. Although commonly used, these approaches have inherent limitations due to potentially incomplete HCP resolution, incomplete transfer to the blotting membrane, a primary reliance on visual comparison, potential inaccuracies in protein counts when modifications result in multiple spots for a single protein, and the use of denaturing conditions that destroy native epitopes, potentially underestimating coverage.^1-5^

More fundamentally, it is unclear how to satisfactorily define adequate percent coverage against the total HCP population found in a given cell culture process. Several authors have analyzed Chinese hamster ovary (CHO) cell culture fluids and identified thousands of expressed proteins,^6-11^ but much smaller numbers of proteins have been identified in downstream samples.^12-18^ Only complete reagent coverage (i.e., 100% immunoreactivity) can ensure all potential HCP impurities can be monitored through downstream operations, but obtaining complete ELISA reagent coverage against the upstream HCP population is not achievable and not a regulatory requirement.^3,19,20^ The dilemma is that if reagent coverage is less than 100%, there can be no assurance that all downstream HCP impurities will be detected. The level of actual risk for any given product is difficult to evaluate because it depends not only on the extent of ELISA reagent coverage, but also the likelihood that immunologically unreactive HCPs persist downstream and have a propensity to cause harm.^19,21-25^

Questions regarding ELISA capability are critical, and regulatory agencies require justification of ELISA suitability for monitoring a particular product and process. Suitability is primarily defined in terms of reagent coverage against the total upstream HCP population, along with considerations of method performance. The onus is on the sponsor to demonstrate suitability of the ELISA reagent for a given product using appropriate experimental methods.^3,26^ Gel-based assessments of HCP expression and ELISA reagent coverage would be strengthened by application of proteomic methods. In particular, the inherent uncertainties of gel-based techniques could be reduced by proteomic analysis of upstream HCP expression, identification of downstream HCP impurities, and protein-specific assessment of ELISA reagent immunoreactivity.

Increasingly, mass spectrometry (MS) is used to identify HCP process impurities. MS characterization has demonstrated sensitive identification of HCP impurities present at low single digit parts-per-million (ppm) levels in purified drug substance,^12-14,17,18,27,28^ identified HCPs with consequences to product stability or patient safety,^22,24^ and been used to monitor clearance of individual HCPs. Proteomic analysis applied to downstream purification and individual unit operations has greatly enhanced understanding of mechanisms for HCP persistence in purified products. In particular, studies have demonstrated correlation between downstream impurities and abundance in harvest fluid,^16,18^ evaluated interactions between HCP and chromatographic resins,^29^ and demonstrated persistence of HCP impurities due to product specific interactions.^15,21,30^ These studies are important for establishing mechanisms whereby downstream purifications fail to clear HCP. However, if it can be demonstrated through proteomic analysis that HCP impurity composition is consistent across lots for a well-controlled process, routine proteomic testing of individual lots may not be justified. In such a case, the ELISA reagent suitability could be rigorously justified by demonstrating reactivity against the downstream impurities identified by MS. In this paradigm, ELISA assay is likely to remain the industry standard method for routine HCP testing for reasons of throughput and simplicity.

Reconciling MS-based HCP impurity identifications with quantitative ELISA results and qualitative immunoreactivity assessments remains a substantial challenge for the biopharmaceutical industry. This is particularly true when quantitative measures of HCP impurity levels obtained by MS and ELISA are discordant, suggesting a persistent impurity is undetected by one of the methods. Industry standard targets for total HCP impurity levels exist, but no safe level for residual HCP has been defined, and it is unlikely all HCP impurities pose the same risks and require the same level of clearance. Identification of individual HCP impurities by MS necessitates development of HCP-specific risk assessment frameworks to guide decisions surrounding acceptable impurity levels.^2,19,20^ Understanding the analytical capability to monitor a particular HCP impurity during routine manufacturing is likely to be a critical aspect of risk assessment in this context.

We developed an approach that can be used to determine ELISA reagent coverage by proteomic analysis following affinity purification (AP-MS) to reduce the level of risk and uncertainty associated with routine ELISA testing. This method can efficiently reconcile the quantitative results provided by ELISA with identification of individual HCP process impurities by MS. A commercial anti-HCP polyclonal reagent was immobilized to magnetic beads and used to affinity purify cognate HCP. The affinity-captured HCP were subsequently identified using traditional bottom-up proteomic approaches. From a single experiment, we were able to identify hundreds of HCPs with immunoreactivity because of their unique presence in immunoaffinity captures (immunocapture) using the immobilized ELISA reagent compared with negative controls. In parallel, we used data-dependent acquisition (DDA) methods to characterize the HCPs present in a null cell harvest fluid, and identified the downstream process impurities present in a therapeutic monoclonal antibody (mAb) following Protein A purification. Together, these data enable: 1) robust and direct assessment of ELISA reagent coverage against the total HCP population expressed in cell culture, analogous to traditional methods, 2) specific assessment of reagent coverage against the subset of HCPs present as actual downstream process impurities, and 3) protein-specific risk assessments that can inform analytical testing strategies and downstream process development. We envision that these methods can be used to develop a holistic understanding of host-cell protein impurity clearance in our products and support the utilization of quantitative ELISA for routine process development and release testing.

## Results

ELISA assays are used to evaluate clearance of HCPs from recombinant biotherapeutics. These assays provide a single quantitative measurement of the total HCP level, but no information about individual impurities that may be present. Similarly, methods for demonstrating ELISA suitability rely on 2D electrophoretic separations that do not identify which HCPs are recognized by the ELISA reagent. Mass spectrometry can be used to identify HCP downstream process impurities and monitor their clearance, but it is challenging to implement in high throughput or regulated environments. We describe an approach to determine ELISA reagent coverage on a protein specific basis using affinity purification and proteomic analysis (AP-MS), providing a means to reconcile MS identification of downstream HCP impurities with quantitative ELISA results. This approach presents substantial advantages for developing a holistic understanding of HCP impurity clearance, analytical capability, and evaluating ELISA suitability against the upstream and downstream HCP populations.

### MS characterization of HCP immunogen

**Figure 1.**
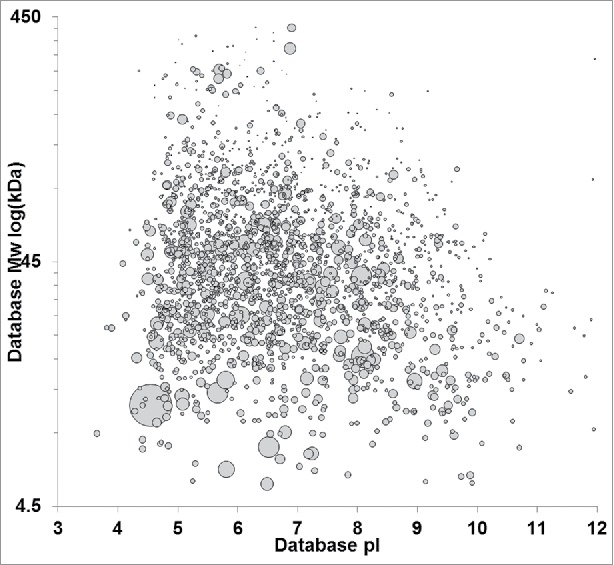
Virtual 2D-PAGE of the HCP immunogen based on MS-protein identifications. Identified proteins were plotted by database molecular weight and pI. spot size is scaled to relative protein abundance as determined by Mascot emPAI. Null cell HCPs span the molecular weight range from ∼5 to 450 kDa with pls ranging from 3–12.

A critical reagent for assessing ELISA suitability is an immunogen that appropriately represents the diversity of host cell expression in the upstream process. Commonly, this immunogen may also be used to develop a “platform” ELISA that can be used for multiple products with sufficiently similar upstream process conditions. Our studies used an HCP immunogen obtained from combined clarified harvest fluids of a null-transfected CHO-DG44 cell line grown under several different upstream process conditions. The immunogen was subjected to trypsin digest and proteomic analysis as described in the methods. Fig. 1 shows a virtual 2D-gel of the null cell protein population according to database molecular weight and pI, where spot size is scaled according to relative abundance. Protein abundance was determined by spectral counting using the emPAI statistic calculated by Mascot. Approximately 2,000 HCP proteins were identified; these proteins spanned database molecular weights from 5.6 to 450 kDa and an isoelectric point (pI) range of 3.7 to 12.0. Our results are consistent with previous reports of HCP expression in high-viability CHO cell cultures that identified ∼1500 HCP proteins.^6-10^ Proteomic analysis of the individual harvest fluids before combination showed a high degree of similarity in HCP expression across the samples. Approximately 80% similarity was observed in the most abundant 1000 proteins. Consistent with previous reports, the greatest source of variability in the samples was increased HCP identifications with increased with days of cell culture, likely attributable to cell lysis as viability decreases (data not shown).

### Affinity purification of HCP

Affinity purifications using a polyclonal reagent and heterogeneous antigen pool pose technical challenges. Antibodies against individual HCPs will be present in different titers and are likely to have a range of antigen binding affinities. Additionally, the HCPs in the immunogen are present at divergent concentrations. Since a bead-based system has finite surface area, coverage determination may be adversely affected by competition for limited binding sites, differences in affinities, or disparate HCP concentrations. For example, abundant HCPs that are recognized by a high titer antibody may effectively out-compete other HCPs for surface binding sites and dominate the immunocapture pool. Similarly, the population of captured HCPs may change over time as proteins recognized by antibodies with high-affinity or low off-rates gradually displace others in a process analogous to the Vroman effect observed in surface binding experiments.^31^ Finally, identification of low-abundance HCPs may not be possible if an insufficient amount of protein is recovered. Consequently, 3 conditions were used for immunogen binding to minimize the possibility that coverage determination would be limited by the particular experimental antigen binding conditions.^32^ We incubated immunogen with primed beads at a saturating HCP load and a sub-saturating load to diversify capture conditions. Immunocomplexation was also performed in solution by incubation of the HCP immunogen and biotinylated capture reagent before bead addition.

**Figure 2.**
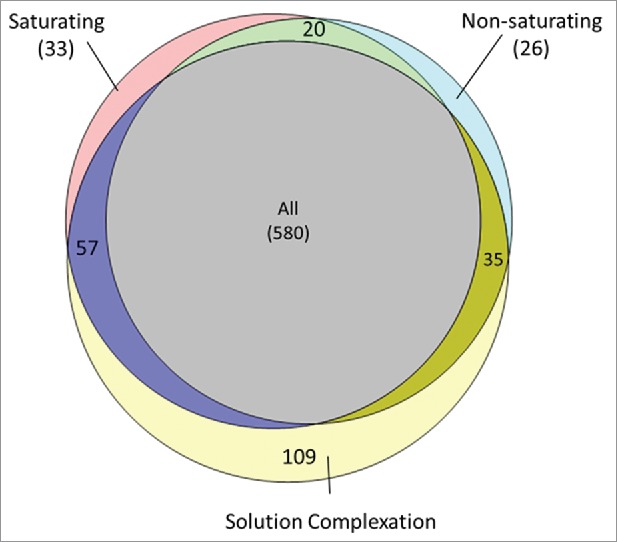
Distribution of HCP identifications among the saturating, non-saturating and solution complexation groups. HCP identification was highly similar between the saturating and non-saturating conditions with few unique identifications to either group (< 4%). Solution complexation before bead addition yielded unique HCP identifications compared with the direct bead binding methods (∼13%).

HCP identifications varied among the different immunogen complexation conditions. Fig. 2 shows the distribution of HCP identifications among the saturating condition, the sub-saturating condition and the solution complexation condition. The majority of HCPs identified were present in all 3 groups (580 proteins, 67%). Protein identifications were highly similar between the saturating and sub-saturating sample binding conditions (96% similarity in protein identification). The difference in protein identification between these groups was on-par with the observed difference in protein identification between replicate injections of a given sample (data not shown). These results indicate that immunogen binding concentration did not have an appreciable effect on HCP identification for the sample and conditions evaluated. In contrast, the solution binding condition had a relatively high percentage of unique protein identifications (109 proteins, 14%) compared with the bead binding groups. This likely arises from differences in immunocomplex formation in solution followed by bead capture compared with complexation on the primed bead surface. These results indicate that solution and bead binding can be combined to ensure diverse immunogen capture, while HCP loading concentrations are less significant.

Affinity purification (AP) experiments for coverage evaluation must be constructed to ensure specificity. A non-specifically interacting HCP could be misidentified as immunoreactive (false positive), and a reactive HCP might be missed due to limitations of the experimental conditions (false negative). The risk of a false positive is particularly undesirable from a process development perspective, since it might result in a decision to monitor a concerning impurity with an ELISA that lacks coverage. Our experiments were designed with this possibility in mind, and included 2 negative controls to ensure specificity. The HCP immunogen was incubated with unprimed beads as well as beads primed with a humanized IgG. The unprimed beads offered a maximum potential for non-specific interactions, while the IgG-primed beads provided a control for potential interactions between HCP proteins and conserved antibody domains. We conservatively defined our threshold for immunoreactivity as unique identification of an HCP only in the anti-HCP purification and neither of the negative controls. This enabled rapid and definitive identification of those proteins with the most certain immunoreactivity, but this approach is expected to understate the true coverage because some proteins with coverage may also be purified non-specifically in the negative controls. This ambiguity can be resolved for individual HCP process impurities of interest by comparison of their abundance between the anti-HCP immunocapture and negative controls, since specific immunoreactivity will increase the protein abundance over background.

**Figure 3.**
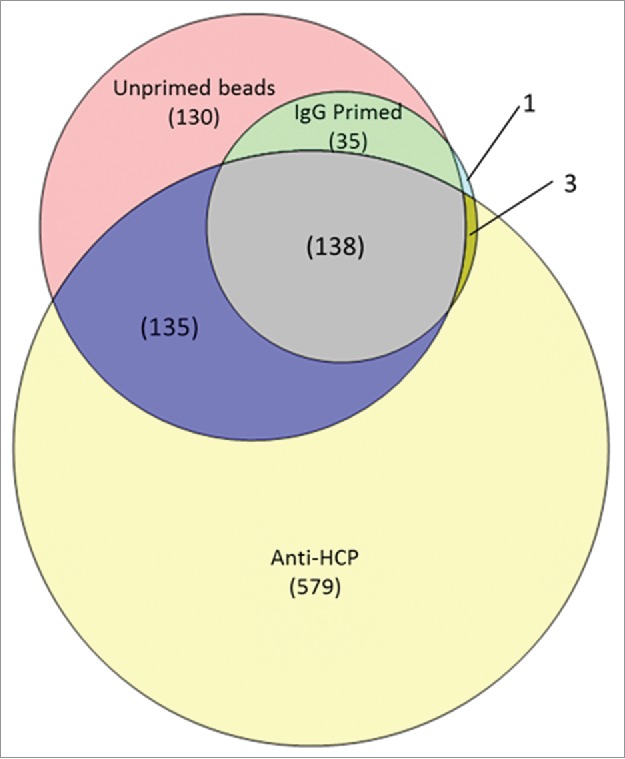
Comparison of HCP protein identifications in the anti-HCP immunocapture compared with the IgG and unprimed bead negative controls. 579 proteins were uniquely found in the anti-HCP immunocapture, indicating specific immunoreactivity with the ELISA reagent. HCPs identified in both the anti-HCP and negative control immnunocaptures have ambiguous coverage and can be analyzed for specific reactivity on the basis of differential abundance.

Differences in protein recovery and identification were observed during AP-MS between the anti-HCP and negative control groups. Purifications using the anti-HCP reagent recovered more total protein for the same sample load than the negative controls using an IgG or unprimed bead, as shown in Table 1. The unprimed bead group had limited protein recovery and the IgG primed beads were intermediate between unprimed and anti-HCP groups. Increased protein recovery in the IgG group is at least partially attributable to elution of the capture IgG, since the capture IgG and bovine serum albumin (BSA) were the most abundant proteins in the IgG control group assessed by spectral counts. Fig. 3 compares protein identifications between the groups. The fewest HCPs were identified in the IgG control (177 total), and 98% of these proteins are also identified in the unprimed control (438 total). These recovery and identification results are consistent with the assumption that diverse non-specific interactions with the unprimed beads result in low-level HCP recovery. Non-specific interactions are largely blocked using the IgG control, reducing the number of HCP identified in this group. The HCPs identified uniquely in the anti-HCP purification are of greatest interest, since these are the proteins with immunoreactivity. Comparing across all binding conditions (saturating, non-saturating, and solution binding), 579 individual HCPs were determined to have immunoreactivity with the ELISA reagent. The AP-MS method efficiently delivers far more information compared with alternative approaches such as 2D-western that rely on recombinant standards or gel excision followed by proteomic analysis for protein identification. Importantly, the immunoreactive proteins identified by AP-MS enable direct and comprehensive assessment of ELISA reagent coverage against either the total HCP population or a subset of downstream HCP impurities on a protein-specific basis.

**Table 1.** Total protein recovery from each affinity purification sample group. Saturating, non-saturating, and solution complexation using the anti-HCP reagent yielded the greatest amounts of protein followed by the IgG negative control bead binding condition and the unprimed negative control beads.

Binding group	Recovered Protein (ug)
Saturating	28.7
Non-saturating	25.4
Solution complexation	26.4
IgG control	17.3
Unprimed control	4.9

### Assessment of ELISA reagent coverage by AP-MS

**Figure 4.**
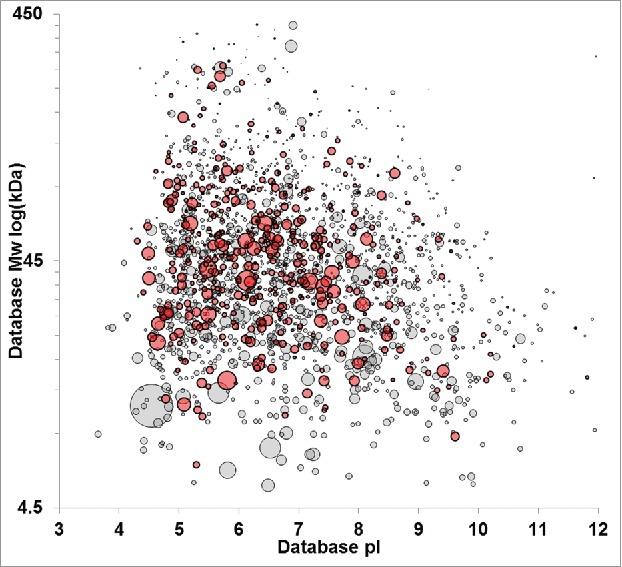
Virtual 2D-PAGE comparing HCPs identified in the null cell immunogen without ELISA coverage (gray) with HCPs that have unambiguous immunoreactivity with the anti-HCP reagent (red) as assessed by unique affinity-purification MS identification relative to negative controls. Proteins recognized by the ELISA reagent span the molecular weight and pI range. Spot size is scaled relative to protein abundance in the immunogen according to Mascot emPAI statistic.

**Figure 5.**
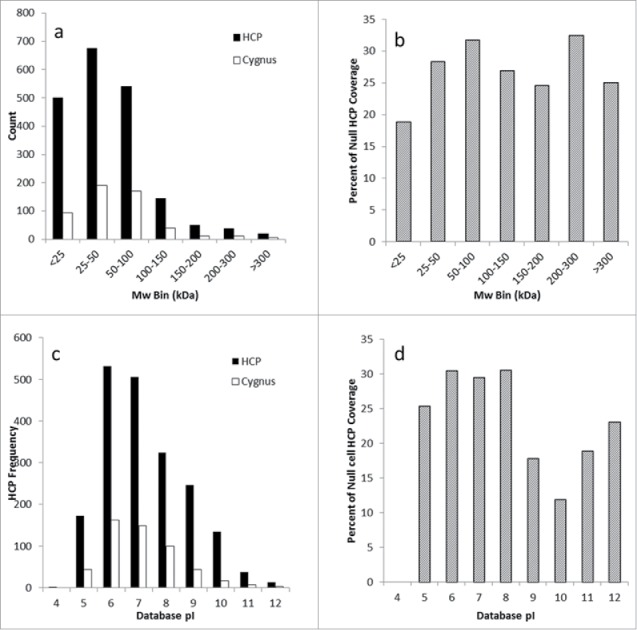
Evaluation of anti-HCP coverage across the molecular weight and pI distribution by protein number and percent coverage. Fig. 5a compares the number of HCPs in each molecular weight bin compared with the number recognized by the Cygnus reagent, and Fig. 5b shows the percent coverage as a function of molecular weight. Fig. 5C compares the number of HCPs identified in each pI bin compared with the number recognized by the Cygnus reagent, while Fig. 5D shows the percent coverage as a function of pI.

The experimental approach we outline enables a shift to protein-specific ELISA coverage assessment that is complementary to downstream HCP impurity identification by MS. However, coverage assessment against the total HCP immunogen population is also possible. Fig. 4 shows a virtual 2D-DIGE plot comparing the HCP immunogen to the immunoreactive HCPs identified by AP-MS. The overall coverage against this immunogen is ∼30% when the number of proteins identified in the immunogen is compared with those identified in the AP-MS. Qualitative visual assessment of Fig. 4 shows the ELISA reagent is immunoreactive across the molecular weight and pI range, indicated by reactivity in every quadrant of the virtual DIGE. The coverage can also be readily assessed on a quantitative basis using the individual HCP identifications shown in the DIGE. Fig. 5a shows the histogram of HCP identifications across the molecular weight range for the immunogen compared with the immunoreactive HCPs, while Fig. 5b shows percent coverage across the molecular weight range. Consistent with visual assessment of Fig. 4, percent coverage is consistent from 25–300 kDa, ranging from 24–35%. A slight decrease in coverage to 18% was observed below 25 kDa, likely reflecting the known difficulties of eliciting effective immunoresponse against low molecular weight HCPs. Similarly, Fig. 5c and 5d show the histogram of HCP identifications and percent coverage across the pI range. Fig. 5d shows consistent coverage of 25–30% across the pI range. A slight decrease in percent coverage is observed above pH 9. The decreased coverage of the basic HCP proteins may simply result from the underlying molecular weight distribution of this population, which is concentrated below 50 kDa (Fig. 1), or it may be a statistical artifact since fewer basic HCP proteins are identified in the experiments compared with the acidic proteins. While the breadth of coverage observed for this ELISA reagent across the immunogen HCP population is good, the absolute coverage is lower than typically desired depending on the stage of clinical development.

### Resolving ambiguous coverage assessments

The standard we used to determine immunoreactivity is highly conservative, requiring unique protein identification compared with both the IgG-primed and unprimed immunocapture groups. Arguably, it would be justifiable to determine immunoreactivity based on comparison to the IgG negative control group only. As indicated in Fig. 3, bead priming with an IgG blocks many non-specific protein-bead interactions, resulting in fewer HCP identifications than purifications with unprimed beads. Priming with the anti-HCP reagent would be expected to have a similar blocking effect as the IgG control. The blocking effect is not accounted for when the unprimed beads are included as a negative control for immunoreactivity. If immunoreactivity were instead defined as unique identification in the anti-HCP group relative to the IgG control only, the number of immunoreactive HCPs would increase by ∼30% (since 135 proteins are shared between the anti-HCP and unprimed groups but absent in the IgG negative control, Fig. 3). However, this standard might increase the risk that an HCP purified non-specifically could be misidentified as having immunoreactivity. Comparison of protein abundance is an alternative approach to determine specific immunoreactivity for HCPs identified in the anti-HCP group and one or more of the negative controls. Immunocomplexation is expected to increase the amount of protein recovered relative to the non-specific background. Such analysis can readily be implemented using software that integrates database search results with peptide quantification. Although it would be feasible to interrogate the abundance of all proteins ambiguously identified in the anti-HCP and unprimed groups for evidence of immunoreactivity, we chose to limit our inquiry to specific proteins known to be downstream process impurities. This focuses analytical effort on the HCPs with the most consequence.

### Application to HCP process impurity analysis

**Figure 6.**
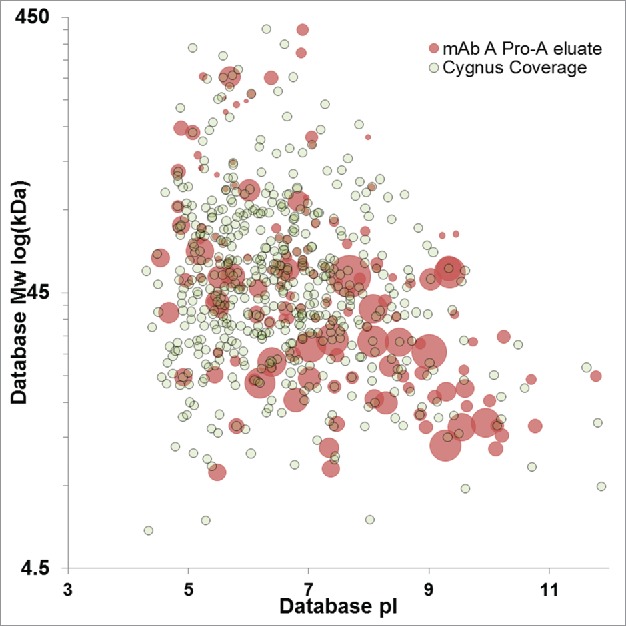
Virtual 2D-DIGE comparison of downstream impurities identified in the Protein A eluate of mAb A (red) and HCPs with ELISA coverage identified uniquely by anti-HCP affinity purification (gray). Protein A impurities with a concentric gray dot have confirmed ELISA immunoreactivity. Protein A impurity spot size is scaled relative to protein abundance, while fixed spot size is used for HCPs identified by anti-HCP affinity purification.

Downstream unit operations provide robust HCP clearance. In the case of therapeutic antibodies, substantial clearance is obtained at the first step of purification using Protein-A chromatography.^15,33^ The limited subset of HCPs that persist through this unit operation are the most critical to monitor by ELISA and provide the most relevant framework for assessing ELISA reagent suitability. A direct assessment of the reagent coverage against actual process impurities can be made by comparing HCP impurities identified in Protein A eluates with protein-specific characterization of ELISA immunoreactivity. HCP impurities were identified in a laboratory-scale purification of a therapeutic mAb (mAb A) using DDA acquisition methods. Approximately 150 HCPs were identified as downstream impurities using this methodology. The top 25 most abundant proteins are summarized in Table 2. Previous publications have characterized at least 15 of these proteins as downstream impurities. The downstream HCP impurity population is shown as a virtual 2D-Gel in Fig. 6. Comparison of Fig. 6 with the immunogen shown in Fig. 1 clearly illustrates the reduction in overall HCP numbers expected from Protein A purification.

**Table 2.** The 25 most abundant downstream impurities assessed by emPAI in Protein A eluate of a therapeutic mAb. Proteins with specific immunoreactivity are noted in the anti-HCP coverage. Proteins described previously in the literature as downstream impurities in antibodies are noted. HCPs with specific immunoreactivity determined by unique identification in the anti-HCP immunocapture are denoted. HCPs with coverage determined by interrogation of differential protein abundance between the positive and negative affinity purifications are also noted using an ^α^superscript. Among the top 25 impurities identified, only 2 lacked ELISA coverage as assessed by affinity purification.

Rank	Protein	Previously Described	Anti-HCP Coverage
1	Pyruvate kinase	Y	Y^α^
2	Glyceraldehyde-3-phosphate dehydrogenase		Y
3	Guanine nucleotide-binding protein subunit β-2-like 1	Y	Y
4	Macrophage metalloelastase (MMP-12)		
5	40S ribosomal protein S3	Y	Y^α^
6	Nidogen-1	Y	Y
7	Serine protease HTRA1	Y	Y^α^
8	Complement C1q tumor necrosis factor-related protein 5	Y	Y
9	40S ribosomal protein S20		Y^α^
10	60S acidic ribosomal protein P0		Y
11	Fructose-bisphosphate aldolase	Y	Y^α^
12	Galectin-3-binding protein	Y	Y
13	60S ribosomal protein L12		Y^α^
14	Elongation factor 1-α	Y	Y
15	Clusterin	Y	Y
16	Actin, cytoplasmic 1	Y	Y
17	Phospholipid transfer protein		Y
18	Peptidyl-prolyl cis-trans isomerase		Y^α^
19	Lactadherin (Fragment)	Y	Y^α^
20	Retinoid-inducible serine carboxypeptidase		Y^α^
21	Lysosomal α-glucosidase	Y	Y^α^
22	GTP-binding nuclear protein Ran		Y^α^
23	Fibronectin		Y^α^
24	Elongation factor 2	Y	Y^α^
25	Heat shock protein HSP 90-α	Y

The discrepancy in protein number between the immunogen and the Protein A eluate illustrates the difficulty of assessing ELISA coverage against the upstream HCP population. Most HCPs present at harvest are not process impurities, and obtaining complete ELISA coverage against the upstream population is impractical or impossible. However, without complete immunoreactivity, there is always a possibility that an HCP could persist through downstream purification and be undetected by ELISA, posing a risk to patient safety or product quality. If ELISA coverage is instead assessed against the downstream protein impurities present in a given product (rather than the complete HCP population), risks can be more rigorously managed. As an example, 105 of the HCP impurities identified in mAb A had reagent coverage in the conservative assessment of the HCP-immunocapture (i.e., unique identification in the anti-HCP group). This corresponds to ∼57% ELISA reagent coverage against the actual process impurities, as illustrated in Fig. 6. Notably, this is 2-fold higher coverage than determined against the total HCP immunogen, and might lead to a different conclusion regarding the suitability of the reagent. Assessing the reagent coverage against the downstream impurities focuses on the HCPs of greatest consequence to the product, enabling a more relevant evaluation of reagent capability. The coverage data that we have presented utilizes the most conservative assessment of immunoreactivity possible from our experimental design. The reagent coverage could be increased by impurity-specific inquiry of HCPs ambiguously identified in both the anti-HCP and negative control immunocaptures. The potential value of this analysis is illustrated in Table 2. Of the 25 HCP impurities in Protein A eluate listed Table 2, only 10 had unambiguous coverage. The remaining 15 were identified in both the anti-HCP immunocapture and negative controls.

**Figure 7.**
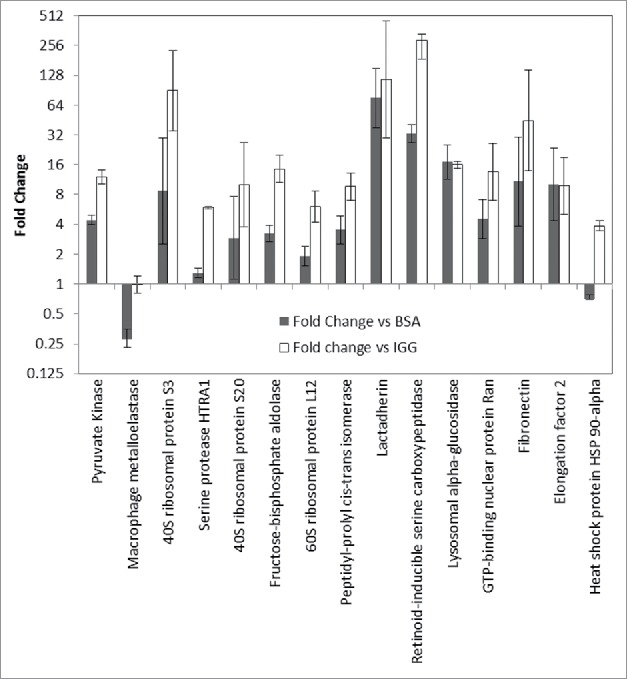
Comparison of HCP abundance in anti-HCP immunocapture compared with IgG negative control (open) and unprimed bead control (solid). Log2 fold change is plotted for each protein. Proteins were deemed to have immunoreactivity if the protein abundance in the anti-HCP immunocapture was greater than in the negative controls.

Evaluation of HCP abundance between the anti-HCP AP and negative controls can identify additional HCPs with reagent coverage. Increased protein abundance in the anti-HCP group is evidence of specific immunoreactivity for a protein that is identified at low abundance in the negative control due to non-specific interactions. Skyline was used to analyze the abundance of the 15 HCP impurities in the Protein A eluate of mAb A that had ambiguous immunoreactivity. These proteins were re-classified as immunoreactive if their abundance in the anti-HCP group was greater than in the BSA and IgG negative controls based on 95% confidence intervals for protein abundance. Fig. 7 illustrates the analysis performed. From this analysis, 13 additional immunoreactive HCP impurities were identified without additional laboratory-based experimentation. The additional HCPs with confirmed coverage from this analysis are noted in Table 2, extending the anti-HCP reagent coverage to 92% of the top 25 impurities found in the Protein A eluate of mAb A. All identified downstream process impurities with ambiguous coverage could be readily analyzed using the same approach

Utilizing the downstream process impurities to guide manual validation of HCPs with ambiguous coverage maximizes the value of the immunocapture while minimizing time spent on validation. This approach would be particularly valuable for a multi-product ELISA, since a single experiment could provide reagent coverage information applicable to many products. As new HCP downstream impurities were identified, their coverage could be directly evaluated against the existing affinity purification data. As apparent in Table 2, some HCP impurities do not have confirmed immunoreactivity even after manual interrogation. This is not unanticipated given that some proteins may not elicit a robust immunoresponse due to sequence homology or other factors. Additionally, a limitation of the immunocapture experiment is that it can confirm immunoreactivity, but not the absence of reagent coverage. The experiment may fail to identify a protein with reagent coverage for several reasons, including low antigen concentration in the immunogen, low antibody titer, loss of a bound antigen in the bead washing steps, failure to elute a tightly bound antigen, or failure to bind an antigen or antibody under the experimental conditions. Additional work, both MS- and gel-based, would need to be undertaken to support a conclusion that an impurity lacked immunoreactivity. It should also be noted that immunogen coverage by the ELISA reagent (as assessed by AP-MS or 2D-western) does not necessarily imply coverage by the ELISA, since HCP assays are commonly performed in a sandwich format that requires 2 epitopes for detection. Despite this limitation, AP-MS evaluation of ELISA reagent reactivity provides valuable information to assess potential gaps in ELISA reagent coverage that can inform further experimental evaluations of ELISA suitability.

## Discussion

Limited protein-specific knowledge surrounding upstream HCP expression and downstream process clearance is a challenging aspect of therapeutic protein development. Anti-HCP ELISAs, which are routinely used to monitor HCP process clearance, provide quantitative estimations of residual HCP levels, offer high throughput, and can be readily implemented in regulated testing environments. However, ELISAs provide no information regarding individual HCP impurities and can be difficult to reconcile with MS-based identifications of downstream HCP impurities. The suitability of a given ELISA for monitoring a particular process must be demonstrated through reagent coverage evaluation, typically against the upstream protein expression. Coverage evaluations involve inherent subjectivity and cannot identify if actual downstream HCP impurities are unreactive. Ongoing advances in mass spectrometry are shifting analysis of HCP away from methodologies focused on consideration of the total host cell proteome to protein- and process-specific frameworks focused on individual protein impurities. Within this framework, anti-HCP ELISA will remain a valuable tool for process development and product release testing if the immunoreactivity of the reagent can be defined on a protein specific basis. The AP-MS approach we describe allows efficient determination of reagent reactivity, complements existing methods of coverage evaluation, and helps establish a holistic understanding of HCP expression, clearance and analytical detectability.

A detailed understanding of HCP expression and process clearance can be obtained by application of proteomics to upstream cultures and downstream purifications processes. A number of recent publications have shown highly similar HCP expression in CHO cell culture using multiple cell lines and variations in process conditions that exceed common variability in biotherapeutic production. Similarly, studies of downstream process clearance have shown that the HCP impurities present in a given product are determined by the nature of their interactions with the product, the chromatographic resins used, the particular downstream processing conditions used, and the abundance of individual HCPs in cell culture. The implication is that for a well-controlled process with limited upstream and downstream variation, the HCP process impurity composition is likely to prove highly consistent from lot-to-lot, and it may be possible to comprehensively identify the HCP impurities for a given process. In this context, protein-specific evaluation of ELISA reagent coverage could provide a rigorous justification of the reagent suitability that is complementary to gel-based approaches, and is highly transferable across products that utilize the same reagent (i.e., a “platform” or “multi-product” ELISA). Perhaps most critically, potential gaps in HCP process clearance could be identified through reagent coverage assessment and downstream impurity characterization, allowing any risks to be mitigated. Impurity-specific risk assessments undertaken when gaps are identified would drive data-driven decisions to guide process or analytical development to best ensure patient safety and product quality and efficacy.

In conclusion, the identification of HCP process impurities by MS increases product knowledge, but cannot be directly reconciled with methods that do not provide protein-specific information, such as ELISA. Despite increasing feasibility of routine MS experiments for HCP identification and quantification, ELISA provides substantial advantages for release testing and support of routine process development. However, the quantitative ELISA result cannot be easily bridged to knowledge of the downstream HCP impurity composition. The AP-MS method described provides a direct, protein-specific assessment of ELISA reagent coverage that provides critical data to reconcile the results of ELISA and MS-based studies of HCP process clearance, and to justify suitability of the ELISA reagent. Unlike traditional gel-based methods of ELISA reagent characterization, AP-MS identifies the specific HCPs that have immunoreactivity and provides an overall reagent coverage assessment across the protein molecular weight and charge distribution. Combined with identification of downstream HCP process impurities, the AP-MS method also enables a reagent suitability assessment relative to the HCPs of greatest potential consequence to the product. Together with emerging MS methods for HCP impurity identification, AP-MS enables a holistic and highly focused assessment of process and analytical capabilities, identification of potential risks, and opportunities for risk mitigation within a protein-centric HCP framework.

## Materials and methods

MAbs were expressed and purified in-house unless otherwise indicated. Laboratory chemicals and reagents were obtained from Sigma-Aldrich (St Louis, MO) unless otherwise noted.

### Antibody biotinylation

Magnetic-streptavidin beads (Thermo Scientific, Waltham, MA) were used to perform immunoaffinity captures because of prior in-house experience with these reagents^34^ and the ability to perform mildly denaturing elution with minimal loss of capture antibody. This approach requires biotinylation of the capture antibodies before bead priming. A commercially available, goat-derived polyclonal anti-HCP ELISA reagent (Product # 3G-0016-AF, Cygnus, Southport NC) and a humanized IgG1 mAb developed at Seattle Genetics were biotinylated with chromophoric biotin according to described previously protocols to achieve an average of 3–4 biotin per antibody. Briefly, antibodies in phosphate buffer were diluted in reaction buffer (500 mM borate, 500 mM NaCl, pH 8.0). SureLINK Chromophoric NHS-biotin (Seracare, Milford MA) in dimethyl sulfoxide was added in 5-fold molar excess. After one hour, the reaction was quenched with glycine and purified by dialysis against phosphate buffer. After purification, samples were analyzed spectroscopically to assess the degree of biotinylation and by size-exclusion chromatography (SEC) to evaluate aggregation and confirm removal of unconjugated biotin. No increase in aggregation was observed for either the negative control IgG mAb or the commercial anti-HCP reagent and no residual free biotin was observed during SEC analysis. Interestingly, the ELISA reagent was found to have ∼23% aggregate under the conditions of the SEC analysis (data not shown). The level of aggregation was unchanged in the biontinylated sample relative to the unmodified reagent.

### Proteomic analysis of HCP immunogen

HCP immunogen was obtained from clarified cell culture fluid and buffer exchanged to phosphate-buffered saline (PBS). A FASP digestion protocol^35^ was applied to prepare the samples using Nanosep Omega spin filters (MWCO 3 kDa, Pall). HCPs were denatured by dilution in 8 M urea at a Rapigest (Waters, Milford, MA) concentration of 0.15% v/v and buffer exchanged to 8 M urea by spin filtration before reduction with dithiothreitol (DTT) at 37 °C for 1 h. Sample alkylation was performed using sodium iodoacetic acid at room temperature after buffer exchange to remove residual DTT. Samples were exchanged into trypsin digest buffer (33 mM ammonium bicarbonate, 1.3 mM CaCl2, pH 7.4) and subject to trypsin digestion overnight at 37 °C using ∼100:1 protein:trypsin w/w. Digests were quenched by addition of trifluoroacetic acid (TFA) to 0.5% and desalted using Thermo C18 100 μL pipette tips (Thermo Scientific, Rockford IL) after removing residual Rapigest by centrifugation. Sample recovery was performed in 60% acetonitrile (ACN) with 0.1% formic acid (FA) and concentrations were determined by bicinchoninic acid (BCA) protein assay. Samples were dried at ambient temperature under vacuum. Sample reconstitution and analysis were performed as described for affinity purified samples.

### Immunoaffinity enrichment optimization

Several aspects of the immunoaffinity enrichment protocol were optimized to maximize identification of HCPs with specific immunoreactivity and to minimize ELISA reagent consumption. Optimal bead priming concentrations and HCP immunogen binding concentrations were determined using BCA assay. For bead priming, the saturating concentration of biotinylated-capture reagent (anti-HCP or IgG1) was determined to be 150 μg/mg bead, while HCP immunogen binding was observed to saturate above 300 μg/mg of primed bead (i.e., no further depletion of HCP could be detected in the flow-through of the primed beads when loaded above 300 μg/mg). Maximum HCP binding was ∼25 μg/mg of primed bead. Several elution conditions were evaluated with a goal of maximizing HCP protein recovery while maintaining selectivity. These experiments showed that HCP protein recovery and selectivity were maximal when HCP was eluted with 10% ACN and 0.1% FA in H_2_O. Using this elution buffer composition, ∼95% of the recoverable protein was obtained in the first elution compared with subsequent elutions using more denaturing buffers. Protein recovery from HCP-loaded primed beads was ∼3x greater than protein recovery from unloaded primed beads using the optimized wash condition. The composition of the protein recovered in harsher wash conditions was not determined, but likely includes a substantial background contribution from the capture antibody and streptavidin bead system. This result indicates that the recovered protein is primarily bound HCP, but background elution of the capture antibody or immobilized streptavidin also contributes to the total protein recovery.

### HCP immunoaffinity purifications

A critical aspect of the experiment is distinguishing specific interactions of anti-HCP antibodies with their cognate antigens from non-specific interactions with the beads or other materials. For this purpose, HCP immunogen binding was performed with anti-HCP primed beads, a negative IgG1 control, and unprimed beads. By comparing HCPs identified in the anti-HCP group to the negative controls, it is possible to distinguish specific interactions. A second critical aspect of the experiment is variation in the conditions of immunocomplexation to ensure that the determination of immunoreactivity is not limited by the experimental conditions. Bead-based HCP immunocomplexation was performed at a saturating concentration of 300 μg HCP immunogen/mg of primed beads and at a sub-saturating HCP concentration of 150 μg HCP immunogen/mg of primed bead. Solution-based immunocomplexation was also performed between the biotinylated anti-HCP reagent and immunogen followed by addition of primed beads, using equivalent antibody, immunogen and bead ratios as the saturating bead binding experiments. All HCP immunocaptures were performed in duplicate. Negative controls in which immunogen was incubated with unprimed or IgG-primed beads, were performed in singlicate. For bead-based binding, the beads were thoroughly washed with PBST, then incubated for 1 h with biotinylated antibody (anti-HCP or IgG control) at 4 °C. For the unprimed control, BSA was added instead of biotinylated antibody. Primed beads were washed with PBST, HCP immunogen was added, and immunogen complexation was performed for 1 h at 4 °C. Following incubation, the beads were washed 3 times with PBS. The composition of the washing buffer is similar to that used in anti-HCP ELISA assays, except for the omission of low concentration polysorbate, which had an adverse effect on MS analysis. Elution was performed on an orbital shaker for 5 min at ambient temperature. For solution binding, the immunogen and biotinylated anti-HCP capture antibody were incubated overnight at 4 °C in PBS. The immunocomplexed solution was subsequently added to washed beads and incubated on a rotisserie for 1 h at 4 °C. PBS washes and elution were performed as for the bead-based binding samples. In all cases, HCP was recovered by evaporation to dryness under vacuum at ambient temperature.

Dried samples were re-suspended in denaturing buffer (6 M urea, 5 mM DTT, 100 mM ammonium bicarbonate pH 8) and incubated for 30 min in a 37 °C water bath. Iodoacetic acid was added to a final concentration of 15 mM and samples were incubated for 30 min at ambient temperature in the dark. Alkylation was quenched with the addition of DTT to a final concentration of 20 mM. Samples were diluted with trypsin digest buffer (33 mM ammonium bicarbonate, 1.3 mM CaCl2, pH 7.4) to a final urea concentration of 1 M. Trypsin was added at 1:40 μg/μg HCP and samples were digested at 37 °C overnight. Digests were quenched by addition of TFA to 0.5% and desalted using Thermo C18 100 μL pipette tips (Thermo Scientific, Rockford IL). Sample recovery was performed in 60% ACN with 0.1% FA and concentrations were determined by BCA. Samples were dried at ambient temperature under vacuum.

### Proteomic analysis of immunoaffinity purifications

Digests were analyzed at the University of Washington Proteomics Resource using nanoflow RP-HPLC on an Orbitrap Fusion. Desalted samples were reconstituted to 0.7 μg/μL in 1% ACN with 0.1% FA by vortexing for 10 minutes, then spun down at 14,000 RPM for 3 min and transferred to autosampler vials. Sample injections targeted 0.7 μg on column. Samples were trapped on a 3 cm × 100 μm Reprosil Pur C18AQ 120A 5 μm column. The analytical column was a custom packed 35 cm × 75 μm column using the same resin. The analytical gradient was 10 to 30% ACN with 0.1% FA over 120 minutes with a flow rate of 3 μL/min. The MS1 scan range was 300–1100 m/z with 120,000 resolution, 50 ms maximum injection time and 4e5 AGC. MS/MS spectra were acquired in the Orbitrap for charge states 2–4 at 15,000 resolution with a maximum injection time of 60 ms, 5e4 AGC target, 5e4 ion threshold, NCE of 29, a 1.6 m/z isolation window and 30s dynamic exclusion.

MS data analysis was performed through Proteome Discoverer version 1.4 using Mascot as the database search engine. Duplicate injections of negative controls or single injections of duplicate preparations for HCP binding conditions were grouped as fractions for data analysis. Experimental spectra were searched against the Uniprot CHO (Cricetulus griseus) database, common contaminants, and the sequence of the IgG control antibody with automatic decoy database search. The database search parameters were full tryptic enzyme specificity with 1 missed cleavage, 10 ppm MS1 mass accuracy, and 0.04 Da MSMS fragment mass tolerance. Carboxymethyl cysteine was set as a fixed modification. Percolator q-values were used for peptide spectral match validation with a 0.01 false discovery rate (FDR) target for strict ID and 0.05 FDR for relaxed ID. Protein identification required a minimum of 2 high confidence peptides (0.01 FDR), counting only rank 1 peptides and a minimum peptide length of 4 residues. Redundant peptides were counted only toward the top scoring protein with strict protein parsimony applied for protein grouping.

### Validation of ambiguous immunoreactivity

Proteins uniquely identified in the anti-HCP immunocapture, compared with the unprimed bead and IgG control bead groups, were determined to have specific immunoreactivity with the ELISA reagent. Proteins identified in the anti-HCP samples and the unprimed beads had ambiguous immunoreactivity. Such proteins could have been purified non-specifically in both groups or could have specific immunoreactivity in the anti-HCP group, as well as non-specific purification in the unprimed group. This would be suspected if the HCP abundance were greater in the anti-HCP group than in the unprimed control. MS1 filtering in Skyline was used to evaluate protein abundance between the anti-HCP immunocaptures and negative controls for evidence of immunoreactivity. Once peptide MS1 transitions were selected for the proteins of interest, Skyline (Skyline-daily, version 3.5.1.9942) was used to calculate log-2-fold changes in protein abundance between the anti-HCP and unprimed bead groups, or the anti-HCP and IgG primed bead groups. At least 2 high quality peptides were used for each protein. Specific immunoreactivity was defined as statistically greater protein abundance in the anti-HCP group than the negative control based on 95% confidence intervals calculated by Skyline. This assessment was based on the experimental variability in the present experiment, but a different standard could be justified based on further experimentation.
